# Tai Chi Chuan optimizes the functional organization of the intrinsic human brain architecture in older adults

**DOI:** 10.3389/fnagi.2014.00074

**Published:** 2014-04-17

**Authors:** Gao-Xia Wei, Hao-Ming Dong, Zhi Yang, Jing Luo, Xi-Nian Zuo

**Affiliations:** ^1^Key Laboratory of Behavioral Science, Institute of Psychology – Chinese Academy of SciencesBeijing, China; ^2^Key Laboratory of Mental Health, Institute of Psychology – Chinese Academy of SciencesBeijing, China; ^3^Laboratory for Functional Connectome and Development, Institute of Psychology – Chinese Academy of SciencesBeijing, China; ^4^Magnetic Resonance Imaging Research Center, Institute of Psychology – Chinese Academy of SciencesBeijing, China; ^5^University of Chinese Academy of SciencesBeijing, China; ^6^Beijing Key Laboratory of Learning and Cognition, Department of Psychology, Capital Normal UniversityBeijing, China

**Keywords:** functional homogeneity, Tai Chi Chuan, meditation, aerobic exercise, executive function

## Abstract

Whether Tai Chi Chuan (TCC) can influence the intrinsic functional architecture of the human brain remains unclear. To examine TCC-associated changes in functional connectomes, resting-state functional magnetic resonance images were acquired from 40 older individuals including 22 experienced TCC practitioners (experts) and 18 demographically matched TCC-naïve healthy controls, and their local functional homogeneities across the cortical mantle were compared. Compared to the controls, the TCC experts had significantly greater and more experience-dependent functional homogeneity in the right post-central gyrus (PosCG) and less functional homogeneity in the left anterior cingulate cortex (ACC) and the right dorsal lateral prefrontal cortex. Increased functional homogeneity in the PosCG was correlated with TCC experience. Intriguingly, decreases in functional homogeneity (improved functional specialization) in the left ACC and increases in functional homogeneity (improved functional integration) in the right PosCG both predicted performance gains on attention network behavior tests. These findings provide evidence for the functional plasticity of the brain’s intrinsic architecture toward optimizing locally functional organization, with great implications for understanding the effects of TCC on cognition, behavior and health in aging population.

## INTRODUCTION

Tai Chi Chuan (TCC) is a form of mind-body exercise that originated in ancient China. In the 1990s, the Western research community began to examine the effectiveness of TCC interventions, using scientific research designs and standardized outcome measures. These studies mainly investigated TCC-induced effects on physical aspects, such as fall prevention, hypertension reduction, and cardiac rehabilitation (Lee and Ernst, 2012) as well as some benefits for mental health, including quality of life, self-efficacy and mood (Caldwell et al., 2009). TCC, a type of complex motor practice and imagination, has also been proven to have positive effects on executive function in older adults. For instance, it was found that TCC has benefits on executive function tasks (Matthews and Williams, 2008). Taylor-Piliae et al. (2006) replicated this result, showing that TCC experts experienced significant improvements during tasks involving the components of executive function (from baseline to 12 months during the TCC program). It is thus widely applied in clinical intervention trials as a type of complementary and alternative medicine (Ding, 2012).

Tai Chi Chuan is also called moving meditation (Robins et al., 2012) because it focuses on the conscious use of slow breath and graceful movement to enhance relaxation and mindfulness. Such mind-body integration exercise has been proven to reshape the patterns of brain structures and functional connectivity (Cahn and Polich, 2006; Luders et al., 2009, 2011, 2012). In particular, the practice of TCC requires a high degree of awareness to maintain an optimum state of effortlessness during the exercise, which poses a great challenge – the mind must control inner thoughts, cultivate mental capability and sustain mindful attention to internal and external sensory stimuli. It is reasonable to postulate that repeated TCC engagement as a form of mental and physical exercise might induce some reliable and optimized changes in brain anatomy and function. As part of the evidence supporting this speculation, a previous report demonstrated significantly thicker cerebral cortices in TCC practitioners compared to healthy age- and gender-matched controls (Wei et al., 2013). Nevertheless, studies of the functional patterns of the brain in relation to TCC practice and its cognitive improvements are still surprisingly sparse.

Relatively few electroencephalography (EEG) studies have reported that TCC can produce changes in mental state or electroencephalogram patterns associated with other alterations of cognitive or physical indices. Field et al. (2010) observed that performance on math computations was significantly improved by a 20-min TCC/Yoga training course and associated with an increase in frontal EEG theta activity. This result has been replicated for TCC in a study of skilled female TCC practitioners that showed a pattern typically occurring during states of relaxation and attention (Liu et al., 2003). While these few studies revealed potentially meaningful patterns of temporal dynamics in the brain under TCC conditions, the spatial patterns of these temporal modes have not been located, especially the temporal synchronizations of brain functional activity within a very small region (i.e., local functional homogeneity or regional homogeneity).

The emergence of resting-state functional magnetic resonance imaging (R-fMRI) makes possible direct examination of the functional architecture of the human brain in high spatial resolution (Biswal et al., 1995, 2010; Zuo et al., 2012). Twenty percent of basal metabolism was delivered to the human brain during rest, while only approximately 5% of this energy was designated to specific activations during cognitive tasks (Raichle, 2010); this is thought to determine the endogenous, or background, neurophysiological processes of the human brain and reflect spontaneous neuronal activity (Raichle et al., 2001; Fox and Raichle, 2007; Zhang and Raichle, 2010). Previous studies indicated that meditation training might change resting-state brain function, focusing on the default network or default mode of the human brain (DMN; e.g., Travis et al., 2010; Brewer et al., 2011; Jang et al., 2011; Taylor et al., 2013). In view of similarities between meditation and TCC on meditative components, these findings provided insights into the brain’s intrinsic connectivity patterns that are induced by meditation. However, the intrinsic functional architecture of the human brain in TCC experts has not been explored.

Accordingly, the present work aims to examine TCC-associated changes in the human brain’s intrinsic architecture and the relevant gains in behavioral performance. Specifically, the functional architecture of the human brain was measured using the functional homogeneity of a small region of the cortical mantle, or 2d surface-based regional homogeneity (2dReHo), which has been demonstrated as an index of temporal synchronizations of functional brain activity (Zang et al., 2004). ReHo was developed in 3d volume space originally (Zang et al., 2004). In this work, we chose its 2d surface-based variant because that 2dReHo reflects the sheet-like nature of cortical organization in its structure and function more appropriately, highly test–retest reliable (Zuo et al., 2013) and more sensitive to detection of disease-related changes of the human brain function (Li et al., 2014). TCC practice has been shown to potentially increase the thickness of brain regions related to motor and executive functions (Wei et al., 2013). Because most structural changes at the macroscopic level result from repeated activations over long periods of training or exercise, it was hypothesized that regionally functional homogeneity of the brain would be different in experienced TCC practitioners compared to controls. It was further speculated that these changes in local functional homogeneity would be related to TCC experience and reflect improved behavioral performance in terms of executive function and cognitive regulation (Szeszko et al., 2000; Colcombe et al., 2004b).

## MATERIALS AND METHODS

### PARTICIPANTS

Forty healthy right-handed participants took part in the section of functional magnetic resonance imaging (MRI) study, including 22 TCC practitioners (age: 52.4 ± 6.8; 7 males, 15 females) and 18 controls (age: 54.8 ± 6.8; 8 males, 10 females) matched for sex, age, and education. Control participants with no physical exercise, yoga, or meditation experience at least 10 years were recruited from the local community, and all TCC practitioners were recruited from local TCC activity centers in Beijing. On average, the participants had 14.6 (SD: 8.6) years of TCC experience, which is defined in this study as the duration of practice. Based on the practice frequency (practice sessions per week) and session durations, the total amount of TCC training per week (intensity) was estimated. The participants’ average TCC training hours per week ranged from 8 to 14 (**Table 1**).

**Table 1:** Participant characteristic.

	TCC experts(*N* = 18)	Healthy controls(*N* = 22)	*p*
Age (years)	52.4 ± 6.8	54.8 ± 6.8	0.26
Gender (males)	7	8	NA
Education (years)	12.2 ± 2.9	11.8 ± 2.9	0.67
TCC duration (years)	14.6 ± 8.6	NA	NA
TCC intensity (hours/week)	11.9 ± 5.1	NA	NA
ICV^1^ (liter)	1.11 ± 0.17	1.12 ± 0.22	0.42
Global ReHo^2^	0.64 ± 0.05	0.65 ± 0.05	0.39
rmsFD^3^ (mm)	0.16 ± 0.09	0.12 ± 0.07	0.16

*^1^ICV: the intracranial volume; ^2^Global ReHo: global mean regional functional homogeneity; ^3^rmsFD: root mean square of frame-wise displacements.*

Written informed consents were obtained from all of the study participants; the study was titled “*The neural correlates of the effects of Taichi on mental health*” and approved by the Institutional Review Board of the Institute of Psychology, Chinese Academy of Sciences (CAS). The ethics committee specifically approved all of the procedures of this study. Before the MRI scans were taken, all of the subjects brought volunteer screening forms to the Institute of Psychology, CAS, and any subjects with histories of hearing or vision problems, physical injury, seizures, metal implants, head trauma with loss of consciousness, or pregnancy were excluded.

### BEHAVIOR TESTS

Ten healthy controls (age: 59.1 ± 2.28; education: 12.1 ± 3.28) and 10 TCC experts (age: 57.1 ± 3.31; education: 12.0 ± 2.58) accomplished behavioral test in this study. Before fMRI scans, the twenty participants completed an attention network test (ANT), which is also called flanker type test to measure different behavioral aspects of attention including alerting, orienting and conflict effect based on the Attention Network theory (Fan et al., 2002). In this study, we only report conflict effect using this paradigm that is target related and reflected the level of executive function. Participants were seated in 65 cm front of a computer screen. Stimuli were presented and responses were collected with E-prime Software 2.0. Participants were instructed to respond as fast and accurately as possible to a target stimulus that was presented in the center of a horizontal row with five stimuli. The target stimulus was an arrow pointing either to the left or to the right and was flanked by two flanker stimuli on each side. Secondly, participants were instructed to press the left mouse button with their left thumb or the right mouse button with their right thumb as fast as possible when the target arrow pointed to the left or right, respectively. The four surrounding flanker stimuli were all arrows pointing tin the same or the opposite direction of the target stimulus or were just neutral stripes. The condition in which all five arrows pointed in the same direction was called congruent target condition. The condition in which the flanker arrows pointed in the direction opposite to the target arrow was named the incongruent target condition. The condition when the four stimuli of flanker were stripes was called the neutral target condition. The target stimulus and the flanker stimuli were presented at a visual angle of 1.1 above or below a cross fixation presented in the middle of the screen.

The target stimulus could be cued in four different ways. In the first cueing condition, an asterisk was presented at the location of the fixation cross (center cue condition) and the target configuration was presented above or below the center of the screen, with equal probability. In the second cueing condition, two asterisks were presented (double condition); the two asterisks were presented at the fixed location of 1.1 of visual angle above and below the center of the screen. Since the cue appeared 500 ms before target onset, the cue provided information on the timing of the target stimulus. In the third cueing condition, an asterisk was presented at the future location of the target stimulus above or below the center of the screen (spatial cue condition). In this case, participants were informed both on the timing and the location of the target configuration. In the fourth cueing condition, no cue was given and, as a consequence, participants had no information about the timing and the location of the upcoming target symbol.

The ANT consisted of one training block with 24 trials and three test blocks with 96 trials each. After the first two blocks, participants took a short break before starting the next one. A single trial consisted of the following: during a variable interval (VI), ranging from 400 to 1600 ms, a fixation cross was presented in the middle of the screen. Then, depending on the cure condition, a cue could be presented for 100 ms. Thereafter, a central fixation was presented for 400 ms, followed by the target stimulus, which was presented for 1700 ms, or shorter if a response was given within 1700 ms. Finally, a fixation cross was presented during a variable delay. The length of this delay was determined by subtracting the reaction time (RT) plus 400 ms from the constant trial duration that was 3500 ms. All 12 combinations of cueing (4) and target (3) conditions were presented in random order within each block. Both RT and error scores were measured for each condition. The level of executive attention is measured by the RT of incongruent condition minus the RT of congruent condition. The conflict effect was calculated on the basis of two measurements: RT and accuracy rate. For the RT, the ratio score of conflict effect was calculated as RT of incongruent condition minus RT of congruent condition divided by mean RT. Only RTs of correct responses were included for the calculation. For the accuracy rate, conflict was calculated as accuracy rate of incongruent condition minus accuracy rate of congruent condition.

### SCANNING PROTOCOL

All of the brain images were acquired using a 3T Trio Tim scanner (Siemens, Erlangen, Germany) with a 12-channel head matrix coil. Resting-state functional images were obtained using an echo planar imaging (EPI) sequence with the following scan parameters: TR = 2000 ms, TE = 30 ms, flip angle (FA) = 90°, slice thickness = 3.0 mm, map = 1.0 mm, field of view (FOV) = 200 mm × 200 mm, and voxel-size = 3.4 mm × 3.4 mm × 4.0 mm. The resulting data included 243 brain volumes with 33 axial slices. During the R-fMRI scans, all of the subjects were instructed to keep their eyes closed, relax and move as little as possible. Importantly, they were required to do not count the number or breath and do not put themselves in meditating state. High-resolution structural images were acquired using a magnetization-prepared rapid gradient echo (MPRAGE) three-dimensional T1-weighted sequence (TR = 2530 ms, TE = 3.39 ms, FA = 7°, voxel-size = 1.33 mm × 1.0 mm × 1.33 mm).

### IMAGE PREPROCESSING

All of the image processing was conducted using the Connectome Computation System (CCS: http://lfcd.psych.ac.cn/ccs.html; Zuo et al., 2013). This pipeline integrates Freesurfer, FSL, AFNI (Cox, 2012; Fischl, 2012; Jenkinson et al., 2012) and in-house Shell/MATLAB scripts to provide a system for multimodal image analysis. The main steps of structural and functional preprocessing in CCS included (1) brain tissue segmentation and cortical surface reconstruction, (2) removal of the first 5 EPI volumes (10 s) from each scan to allow for signal equilibration, (3) slice timing correction, (4) 3D motion correction, (5) 4D global mean-based intensity (10,000) normalization, (6) removal of 26 nuisance covariates of WM/CSF signals and 24 motion parameters estimated with the Friston-24 model (Satterthwaite et al., 2013; Yan et al., 2013), (7) band-pass temporal filtering (0.01–0.1 Hz), (8) removal of linear and quadratic trends, (9) co-registration between individual functional and anatomical images using a rigid boundary-based transformation (BBR; Greve and Fischl, 2009), and (10) projection of functional images onto the standard cortical surface (subject *fsaverage5* in Freesurfer 5.1).

Several covariates were estimated during the above image preprocessing steps. The intracranial volume (ICV) was measured using the Freesurfer segmentation pipeline. The amount of regional volume change needed to warp a subject into the standard surface *fsaverage5* was measured using the vertex-wise covariate, which was derived from the Jacobian determinant of the spherical transform (JAC) to account for heterogeneous changes in regional volume. The warp distortion amount for BBR-based function-to-structure realignment was the minimal cost of the co-registration (mcBBR). The covariate of head motion was measured using the root mean square of the frame-wise displacement (rmsFD; Power et al., 2012; Patriat et al., 2013).

### INDIVIDUAL-LEVEL FUNCTIONAL HOMOGENEITY ON THE CORTICAL SURFACE

**FIGURE 1:**
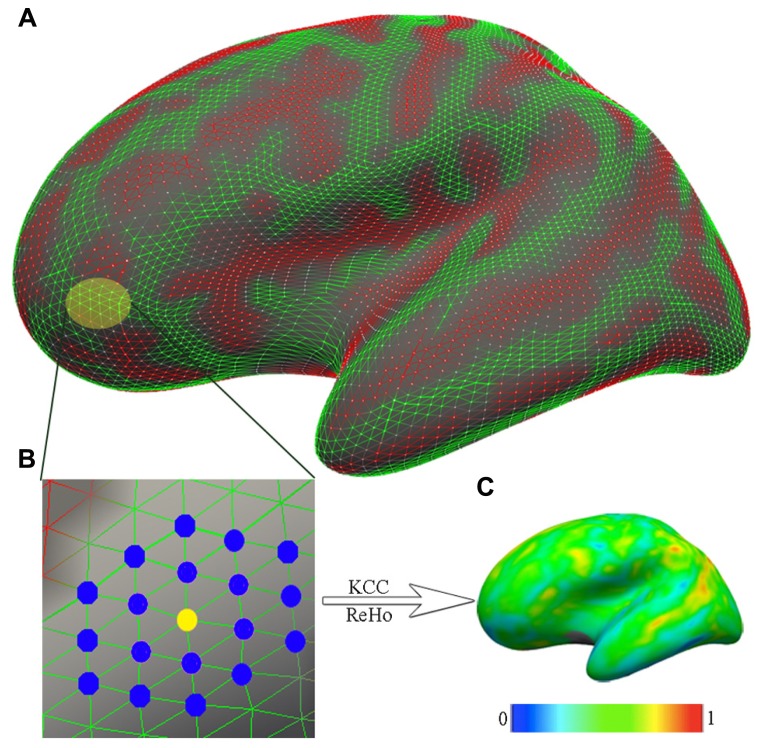
**The computation of functional homogeneity on the cortical surface**. **(A)** The geometry of the cortical surface (e.g., here, the lateral cortical surface of the left hemisphere). **(B)** The preprocessed R-fMRI data are projected onto vertices of the cortical surface. For a given vertex (yellow node), which is indicated as the light yellow patch in **(A)** and magnified in **(B)**, the nearest neighbors are colored blue. Based on R-fMRI time series from all 19 vertices, the Kendall’s coefficient of concordance (KCC) of the center vertex is calculated as the regional homogeneity (ReHo). **(C)** Such calculations are repeated for all vertices on the surface to produce individual vertex-wise KCC-ReHo surface maps. The color map indicates the intensities of the KCC values.

To quantify the regional functional homogeneity of resting-state functional fluctuations in BOLD activity on the cortical mantle, 2dReHo was employed (Zuo et al., 2013). Specifically, for a vertex on the *fsaverage5* surface grid, its 19 nearest neighbors was identified (**Figure 1**), and the Kendall’s coefficient of concordance (KCC) was computed (Zang et al., 2004) for the 19 time series to quantify this vertex’s 2dReHo. This computation procedure was repeated for every vertex on the surfaces of both hemispheres to produce individual 2dReHo surfaces. The global mean of the local functional homogeneity was also calculated as the average 2dReHo across the entire cortical surface for each individual.

### GROUP-LEVEL FUNCTIONAL HOMOGENEITY STATISTICAL ANALYSIS

To test the differences in functional homogeneity between TCC practitioners and healthy controls, a second-level two-sample *t*-test was performed on 2dReHo maps in a vertex-by-vertex manner by controlling for gender, age, ICV, rmsFD, mcBBR, and vertex-dependent JAC. To find regional changes in functional homogeneity, the global 2dReHo was also included as a covariate in the model. A threshold of *p* = 0.01 at the vertex level was used to define clusters for a final cluster-wise correction (*p* = 0.05, corrected). The same test was performed on global 2dReHo, using SPSS 19 (SPSS, Inc.) to examine the global changes in functional homogeneity associated by the practice of TCC.

#### Statistical analysis

Two-sample *t*-test in SPSS was conducted to analyze demographic, behavioral, and global functional homogeneity statistics involving 22 practitioners in TCC group and 18 healthy controls. Using SPSS, we also calculated partial correlation controlling for gender, age, and education between demographic data, behavioral data, and functional homogeneity with significant difference. And only 21 TCC practitioners were involved in the correlation between TCC experience and functional homogeneity for an outlier in practice hours each week. During the correlation of ANT behavioral performance and functional homogeneity, only 9 practitioners in TCC group were involved in the test for the above reason.

## RESULTS

### DEMOGRAPHIC CHARACTERISTICS

The demographic characteristics of all the subjects in each group for functional MRI study are summarized in **Table 1**. The two-sample *t*-tests showed that there were no significant differences in age [*t*(38) = 1.149; *p* = 0.258], gender [*t*(38) = -0.807; *p* = 0.425], or years of education [*t*(38) = -0.435; *p* = 0.666] between the TCC practitioners and the healthy controls. The two-sample *t*-tests between TCC group and control group participating in the behavioral study also did not show any significant difference in age [*t*(18) = 1.571; *p* = 0.134], gender [*t*(18) = -1.852; *p* = 0.081] or years of education [*t*(18) = 0.076; *p* = 0.940].

Regarding the RTs on the ANT test, the TCC group exhibited shorter [though not significantly so; *t*(18) = 1.227; *p* = 0.236] mean RTs relative to the control group in terms of executive function performance. The two groups did not show significant differences in the accuracy of their ANT performances [control group: 99.0 ± 0.8%; TCC group: 99.5 ± 0.6%; *t*(18) = -1.421; *p* = 0.173]. A correlational analysis of the TCC group showed that executive attention performance is negatively correlated with TCC experience (*r* = -0.659; *p* = 0.038).

### DIFFERENCES IN FUNCTIONAL HOMOGENEITY BETWEEN TCC AND CONTROL GROUPS

**FIGURE 2:**
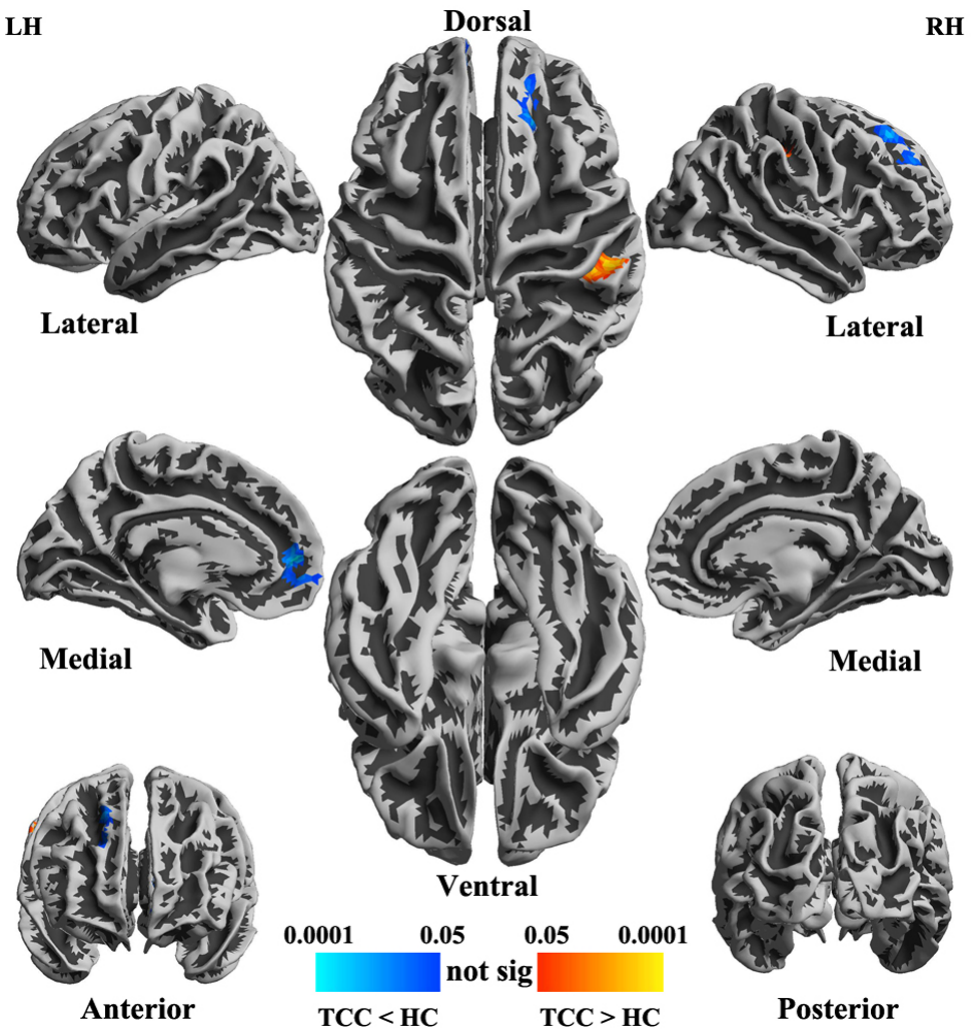
**Statistical maps (*p*-value corrected) of cortical surfaces showing group differences in functional homogeneity measured by 2dReHo in *fsaverage5* standard space**. In TCC experts (compared to healthy control subjects), blue colors indicate decreases in 2dReHo, while red colors indicate increases in 2dReHo.

The two groups (TCC and healthy control) did not differ in global functional homogeneity as measured by 2dReHo (*p* = 0.39; **Table 1**). As shown in **Table 2** and **Figure 2**, significant decreases in 2dReHo were detected in the left anterior cingulate cortices (ACC) and the right superior frontal cortices (SFC) of the dorsal lateral prefrontal cortices (DLPFC) of TCC practitioners compared to the controls. In contrast, increases in 2dReHo were observed in the right post-central gyruses (PosCG) of TCC experts relative to the controls.

**Table 2:** Cortical areas with significant changes in functional homogeneity induced by TCC practice.

				Talairach coordinates (Peak)		
Brain regions	Cortical hemisphere	Brodmann area (BA)	Cluster size (mm^2^)	*X*	*Y*	*Z*
Post-central gyrus (PosCG)	Right	BA2	359.15	43.3	-24.9	36.1
Anterior cingulate cortex (ACC)	Left	BA32	445.78	-12.3	41.9	-4.7
Superior frontal cortex (SFC)	Right	BA9	361.68	17.4	39.1	37.8

### MIND-BODY-BRAIN ASSOCIATIONS

**FIGURE 3:**
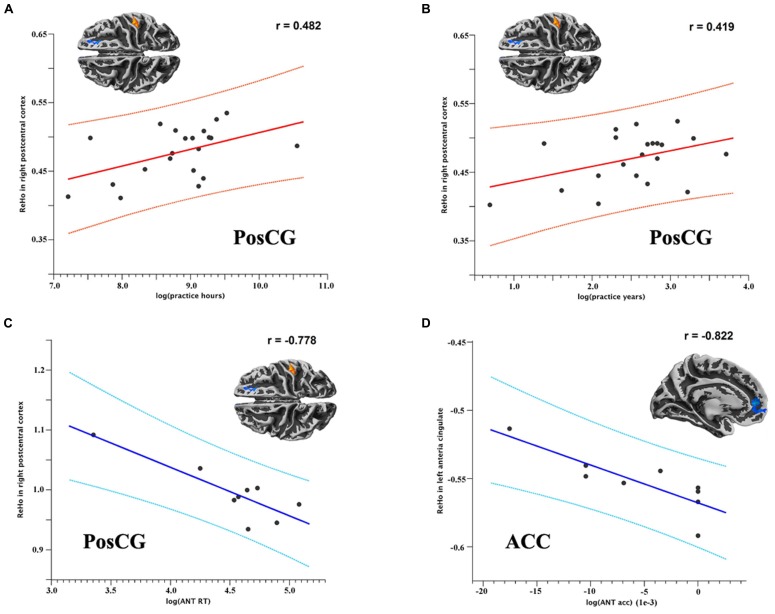
**Scatter plots **(A,B)** show the (marginally) significant correlations between 2dReHo in the right PosCG and TCC practice hours and years, respectively**. Scatter plots **(C,D)** show the (marginally) significant correlations between 2dReHo and reaction time and accuracy on the ANT, respectively. (ANT RT = reaction time on the ANT; ANT acc = accuracy on the ANT).

Since one subject practiced TCC for at least 30 h each week, which is an outlier in distributed scatter plot of descriptive data, we removed this subject from the group and left 21 TCC practitioners being involved in analyzing the correlation between behaviors/descriptive data and functional homogeneity. Within the TCC group, the degree of functional homogeneity of the right PosCG was positively correlated with log-transformed TCC practice hours (*p* = 0.043, *r* = 0.482; **Figure 3A**) and marginally correlated with years (*p* = 0.083, *r* = 0.419; **Figure 3B**). Interestingly, these changes in functional homogeneity tended to negatively correlate with the RTs on the ANT (*p* = 0.069, *r* = -0.778; **Figure 3C**). In addition, the left ACC showed a significant negative correlation between its 2dReHo amplitude and log-transformed accuracy on the ANT (*p* = 0.045, *r* = -0.822; **Figure 3D**).

## DISCUSSION

This is the first study of the intrinsic functional architecture of the human brain in experienced TCC practitioners. Beyond the recently demonstrated regional changes in brain morphometry associated by TCC (Wei et al., 2013), significant differences were observed between TCC experts and the control subjects in terms of locally functional organization as well as in their cognition and behavioral associations. Specifically, the TCC experts exhibited greater functional homogeneity in their right PosCGs and lower functional homogeneity in their right DLPFCs and left ACCs than control subjects. The enhancement of functional homogeneity in the low-level sensory motor region across individuals was associated with individual differences in the TCC experience. The gain in functional integration was significantly correlated with cognitive performance (on the ANT) in TCC experts. Within the high-level brain area (ACC), the gain in functional specialization was also correlated with ANT performance. The value of TCC practice for human cognition, behavior and health, as well as its implications for intrinsic brain functional architecture, will be discussed in the following section.

### TCC OPTIMIZES FUNCTIONAL INTEGRATION OF THE SENSORY MOTOR AREAS

Tai Chi Chuan practitioners showed greater functional homogeneity in their right PosCG than the controls did, indicating improvement in the integration of the somatosensory and motor functions attributed to this area. This region developed early in human evolution across the cortical mantle (Glasser and Van Essen, 2011) and is part of the primary somatosensory cortex, playing key roles in somatosensory perception, such as the localization of touch, two-point discrimination and proprioception. These functional aspects are highly integrated, as observed in TCC practice, and are reflected in improvements in proprioception and balance control. Kerr et al. (2008) adopted a blinded assessor to compare TCC practitioners’ and control subjects’ abilities to discriminate between two different orientations (parallel and horizontal) across different grating widths at the fingertip. They observed that TCC practitioners had superior tactile acuity.

An alternative explanation for the increases in functional homogeneity is that it is possibly associated with improvement in motor balance function. Regarding the characteristics of TCC movement, basic TCC exercise is composed of a series of graceful motions integrated in a continuous sequence so that the body is continuously shifting from one foot to another, with a low center of gravity, which develops the ability to balance. Previous studies consistently reported that long-term TCC practice improved balance control in elderly populations when there was increased reliance on the visual and vestibular systems during stance (Tsang et al., 2004; Wong et al., 2009). TCC participants who showed improvements on measures of functional balance at the intervention endpoint had significantly reduced risks of falling during a 6-month period of post-intervention compared to those in the control condition (Li et al., 2004). The current findings might provide neural evidence for this feature of TCC – that it is capable of improving bodily balance and preventing falls.

Within the PosCG, more experience with TCC practice was associated with greater functional homogeneity. This might reflect the adaptive consequences of TCC training, adding a direct link between the gain in functional integration and TCC practice. Previous studies have repeatedly reported that plasticity of the primary somatosensory cortex could occur during intensive exercise or training. It’s worth mentioning that several decades ago, the association of this region with long-term skills learning in humans was confirmed. For instance, Grafton et al. (1995) found that sequence learning could induce functional change in the post-central cortex. Similarly, Floyer-Lea and Matthews (2005) demonstrated that activation of the post-central cortex was enhanced by 3 weeks of motor skills training. More findings from stroke patients and experimental stroke animals showed that motor recovery after stroke has been associated with reorganized neural activity in this region (Dijkhuizen et al., 2001). Taken together, these findings suggest that the functional homogeneity alteration pattern in the post-central cortex is tightly associated with the integration of higher-order motor execution, such as movement organization, anticipation, and internal representation of actions.

### TCC OPTIMIZES THE FUNCTIONAL SPECIALIZATION OF THE ATTENTION CONTROL AREAS

These results indicate that TCC experts may have more optimal functional homogeneity within the ACC, reflecting improvement of functional specialization (i.e., decreased functional homogeneity) and underlying cognition and behavior. The ACC is thought to play a role in a wide variety of functions, such as error detection, task anticipation, attention (Weissman et al., 2005), motivation, and modulation of emotional responses (Bush et al., 2000). This area contains a cognitive subdivision (dorsal) and an emotional subdivision (ventral). The dorsal part of the ACC (dACC) is connected to the prefrontal and parietal cortices as well as the motor system and the frontal eye fields (Posner and DiGirolamo, 1998), making it a central hub for the top-down and bottom-up processing of stimuli and assigning appropriate control to other areas. In contrast, the ventral part of the ACC (vACC) is connected to the amygdala, nucleus accumbens, hypothalamus, and anterior insula and is involved in assessing the salience of emotions and motivational information. As a high-level association brain area, the ACC is required to be highly specialized at the local micro-scale level and to segregate the actualization of different functional aspects. This may provide an explanation for why less functional homogeneity was detected in this region among TCC practitioners.

Decreases in functional homogeneity were also observed in another association cortex – the superior frontal gyrus – belonging to the DLPFC. This finding replicated previous work on TCC-associated brain morphometry changes in this region using the same samples (Wei et al., 2013). The superior frontal gyrus, which roughly corresponds to Brodmann area 9, belongs to the DLPFC and serves as the high-level cortical area responsible for motor planning, organization, and regulation. It plays an important role in the integration of sensory and mnemonic information and the regulation of intellectual function and action. Almost all complex mental activities require the additional cortical and subcortical circuits connected with the DLPFC. During TCC practice, the DLPFC is possibly called upon for a highly functional assignment and specialization to regulate the whole sequence of interactions and complex movements. Further, the DLPFC is suggested to be sensitive to training and learning tasks that involve multiple processes of cognitive control, such as TCC, reflecting improvements in this region as “flexible hubs” for adaptive task control (Cole et al., 2013).

In bringing the ACC and the DLPFC together, decreased functional homogeneity in TCC experts possibly reflects gains in both task-set maintenance and moment-to-moment tasks in the executive control areas of the attention system (Petersen and Posner, 2012). Previous TCC studies demonstrated that TCC might improve cognitive control skills, such as movement initiation, distraction inhibition and error monitoring, after repeated TCC (Chang et al., 2010). Indeed, related studies confirmed that cognitive function, especially executive control, significantly improved in the elderly following TCC practice (Matthews and Williams, 2008; Taylor-Piliae et al., 2010). It is supposed that executive control is susceptible to TCC training, in multiple ways.

### TCC-ASSOCIATED FUNCTIONAL REORGANIZATION PREDICTS BEHAVIORAL PERFORMANCE

As expected, the ACC was observed to be significantly associated with ANT performance in this study, which indicated that decreased functional homogeneity of the ACC might be related to improvements in attention regulation and cognitive control after long periods of TCC practice. It is well recognized that the primary function of the ACC is to detect conflict and provide cues to other areas in the brain to cope with conflicting control systems. Numerous studies have reported the role of the ACC in cognitive tasks involving monitoring conflicts among competing response tendencies (Gehring et al., 1993; Botvinick et al., 1999). This ability is also regarded as an important profile of executive function to inhibit distractions and control irrelevant things. In particular, recent studies on meditation demonstrated the role of the ACC in improving cognitive regulation during a short-term meditative state. For instance, Tang et al. (2009) observed that a 3-h mental training session increased ACC activity and improved self-regulation as well as increased fiber integrity in the left ACC was induced by an 11-h meditation session (Tang et al., 2010). Cahn and Polich (2006) summarized EEG, ERP, and neuroimaging studies to demonstrate the importance of ACC activation as a marker of increased attentional focus in meditative states. Alternatively, evidence from the domain of aerobic exercise also supports this explanation (Taylor-Piliae and Froelicher, 2004). A longitudinal study showed that aerobically trained older adults showed reduced ACC activity after a 6-month period of walking training compared to controls (Colcombe et al., 2004a). Typically, clinical evidence from patients with first-episode schizophrenia has shown that significantly reduced ACC volume is correlated with executive dysfunction (Szeszko et al., 2000), suggesting the critical role of the ACC in executive function.

Decreases in functional homogeneity were also observed in another brain region associated with executive control – the DLPFC. Although a significant correlation between these changes in functional homogeneity and ANT performance were not observed, a previous study on the same sample detected that TCC practitioners showed better executive control, as well as thicker cortices in this region of this DLPFC, than healthy controls (Wei et al., 2013). It is thus cautiously believed that the decreased functional homogeneity in the DLPFC might be related to gains in executive control performance. It is well documented that the anatomical structures and functional patterns of the DLPFC could be influenced by aerobic exercise and associated with improvements in executive control behaviors. Previous functional MRI studies have consistently found that the effects of aerobic exercise on brain function are mainly on the DLPFC and, simultaneously, improvements in executive control performance. For example, the left DLPFC was more activated and improved (in terms of cognitive performance) by a Stroop task when a group of young adults completed 10 min of acute exercise than when no exercised was performed (Yanagisawa et al., 2010). Evidence from older adults and children also suggests that the role of the DLPFC in executive function is enhanced following aerobic training (Kramer et al., 1999; Davis et al., 2011). The reasonable explanation is that the underlying mechanisms of executive processes supported by the prefrontal cortex, such as multi-tasking, planning, and inhibition, could benefit from aerobic training (Colcombe et al., 2004b).

### LIMITATIONS AND FUTURE DIRECTIONS

Some limitations should be kept in mind when interpreting these findings. First, the two groups of participants may differ in some respects (personality traits, lifestyles, etc.). A cross-sectional study design could not completely exclude the confounding effects of nature or nurture on functional homogeneity in the two groups. In future studies, a longitudinal study of the TCC-brain relationship is needed to rule out these potential confounds. Second, given the relatively small sample size, the results of the present study should be interpreted with caution until they are replicated using a large sample. Future research will test this hypothesis by examining the functional connectivity between these two brain regions in a large sample of TCC practitioners. Third, it is possible that the state of rest may have differed qualitatively between the two groups. It is well known that experienced meditators (and the same might be true for TCC practitioners) automatically engage in their unique (meditative) mental excises, especially if instructed to relax. Thus, we instructed participants to “not meditate” during the resting-state functional MRI scans. We also included a questionnaire to record self-report mood and thoughts during resting-state scans, and found there is no difference between two groups. We thus argue that the measured difference in “the local functional homogeneity” is a long-term consequence of the TCC practice, and not simply due to the different mental activities performed during the scanning procedure. Finally, both physical and mental exercises are involved in TCC and thus our findings can only be read as the overall influence of the two on the brain activity. In future, coming with well-designed and separable behavioral or cognitive tests between physical and mental influences, we could be able to investigate how physical and mental activity complement or interfere with one another.

## CONCLUSION

This study demonstrates that old individuals with extensive TCC training exhibit significant differences in regional homogeneity of brain function relative to TCC-naïve controls. TCC training induces reduced regional homogeneity within both the DLPFC and the ACC and enhanced regional homogeneity in the PosCG. Reductions in regional homogeneity within the DLPFC and ACC may indicate optimized functional heterogeneity or segregated high-level multimodal regions in the executive control-related cortex. In contrast, the PosCG, which is directly related to primary sensory motor information processing, is improved in its functional homogeneity, likely reflecting the improved functional integration offered by TCC. These findings may suggest that resting-state fMRI can be an appropriate approach to studying the effects of TCC on the intrinsic functional architecture of the human brain. Specific regional homogeneity is likely a contributor to improvements in both the motor sensory and executive control behavioral characteristics of TCC and supports the hypothesis that this functional pattern is a possible explanation for the optimization of the high-level cognitive functions observed in TCC. These findings provide neuroimaging evidences that TCC can potentially improve the brain function and cognitive performances in old population.

## Conflict of Interest Statement

The authors declare that the research was conducted in the absence of any commercial or financial relationships that could be construed as a potential conflict of interest.
